# FKH-6 is expressed in male gonadal cells during L3 larval development

**DOI:** 10.17912/micropub.biology.000079

**Published:** 2018-12-31

**Authors:** Aaron Crosby, Mary B Kroetz

**Affiliations:** 1 Department of Biological Sciences, University of South Alabama, Mobile, AL, USA

**Figure 1:**
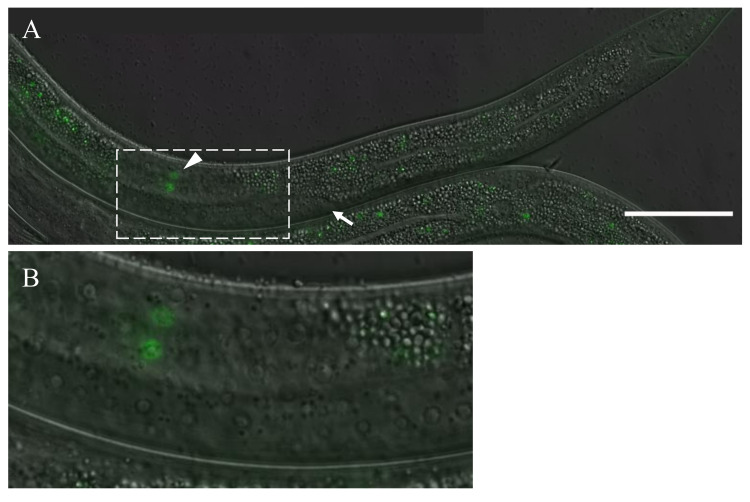
FKH-6 is expressed in the male gonad during the L3 larval stage. Differential interference contrast (DIC) and fluorescence micrographs of FKH-6 expression in the male gonad. Arrow indicates distal end of gonad. Arrowhead indicates GFP-expressing nuclei. Boxed region in A is magnified in B. Bar, 50 m.

## Description

FKH-6 is a forkhead transcriptional regulator that is necessary for proper gonadal development in *C. elegans*. A previous expression study using a transcriptional reporter showed that the *fkh-6* transcript was initially expressed in the gonad from mid-L1 larval stage until around the L1/L2 molt in both sexes (Chang *et al*. 2004). Transcriptional reporters and partial gene fusions indicated that FKH-6 was also expressed from the L3 larval stage through adult in the hermaphrodite gonad in the spermatheca and sheath cells (Hope *et al.*2003, Chang *et al*. 2004). In this current study, the endogenous locus for *fkh-6* was C-terminally fused with GFP using CRISPR-Cas. In addition to recapitulating previously reported expression patterns, this new construct revealed expression of FKH-6::GFP in 2-4 male gonadal cells in the L3 larval stage. This is the first report of FKH-6 expression in males after the L1/L2 molt. The expression of FKH-6::GFP in the gonad of the L3 male is reproducible but short-lived, and coincides with the period when the proximal region of the migrating male gonad is extending past the distal tip of the gonad. This work shows that FKH-6 is expressed in both sexes during the early mitotic period of the gonad in L1 as well as the late mitotic period of the gonad in L3.

## Reagents

The *fkh-6::gfp* fusion was generated using CRISPR-Cas (Dickinson *et al.*2015). Sequencing confirmed the integration of GFP at the 3’ end of the *fkh-6* locus prior to the stop codon. The MBK9 strain *(fkh-6(ndz1[fkh-6::gfp::3xflag]) II; him-8(e1489) IV* was used for imaging. Differential interference contrast (DIC) and fluorescent images were acquired on a Zeiss Axiovert 135 microscope using a Lumenera Infinity 3S-1UR monochrome camera and Infinity Analyze and Capture software.
